# Current Understanding of SIRT7 Function and Its Emerging Roles in the Central Nervous System

**DOI:** 10.3390/cells15060548

**Published:** 2026-03-19

**Authors:** Yuchen Jiao, Chuangui Wang, Shengping Zhang

**Affiliations:** Biomedical Research Institute, School of Life Sciences and Medicine, Shandong University of Technology, Zibo 255000, China; 18392584614@163.com (Y.J.); cgwang24@sdut.edu.cn (C.W.)

**Keywords:** SIRT7, deacetylase, post-translational modification

## Abstract

SIRT7is an NAD^+^-dependent deacetylase predominantly localized in the nucleolus, where it plays important roles in chromatin regulation, transcriptional control, and cellular stress response. Accumulating evidence has revealed that SIRT7 participates in multiple molecular processes, including ribosomal RNA transcription, histone modification, DNA damage repair, metabolic regulation, and inflammatory signaling pathways. Through these mechanisms, SIRT7 contributes to the pathogenesis of various human diseases, particularly cancer and metabolic disorders. In recent years, emerging studies have begun to uncover the roles of SIRT7 in the central nervous system (CNS). Although research in this area remains limited, available evidence suggests that SIRT7 may be involved in neuronal homeostasis, glial function, neuroinflammation, and responses to brain injury. Furthermore, dysregulation of SIRT7 has been implicated in CNS-related pathologies. In this review, we summarize the understanding of SIRT7 molecular mechanisms and its implications in human disease, with special emphasis on its emerging roles in the CNS. We also address unresolved questions and propose future research directions to facilitate a deeper understanding of SIRT7 in neurological physiology and pathology.

## 1. Introduction

Sirtuins (SIRT1–SIRT7) are a family of NAD^+^-dependent deacetylases that function as key metabolic sensors linking cellular energy status to epigenetic regulation and genome maintenance. By coordinating chromatin-based regulatory programs with genome stability pathways and nutrient-responsive metabolic adaptation, sirtuins play central roles in preserving cellular homeostasis [1]. Accordingly, dysregulation of sirtuin activity has been implicated in a broad spectrum of chronic and age-related diseases, including metabolic and cardiovascular disorders, cancer, persistent inflammatory conditions, and tissue dysfunction [2]. In addition, accumulating evidence supports a role for sirtuins in neuroprotection across both acute and chronic neurological disorders, particularly in the context of ischemic injury [3] and neurodegenerative diseases [4].

SIRT7 is one of the least studied members of the sirtuin family. However, studies over the past decade have demonstrated that SIRT7 plays important roles in multiple key biological processes, including the regulation of ribosome biogenesis and RNA polymerase I-mediated transcription [5], histone deacetylation (such as H3K18Ac) [6], and the maintenance of DNA damage repair and genomic stability [7]. In addition, SIRT7 exerts pro-tumorigenic or context-dependent regulatory functions in tumor initiation and progression. Furthermore, SIRT7 is closely associated with metabolic regulation, aging-related processes, and the development and progression of cardiovascular and liver diseases [8]. However, the role and underlying mechanisms of SIRT7 in the nervous system remain unclear.

Although SIRT7 has been established as an NAD^+^-dependent deacetylase, its enzymatic properties remain incompletely characterized. The catalytic activity of SIRT7 appears to be highly context-dependent and comparatively weak under standard in vitro conditions [9], suggesting that additional cofactors or specific nuclear environments may be required for full activation. Whether SIRT7 possesses broader deacylase activities beyond deacetylation and how these activities contribute to cellular regulation remain important questions for future investigation. In parallel, the endogenous substrate repertoire of SIRT7 is still poorly defined. This incomplete substrate landscape substantially hampers a full understanding of SIRT7-mediated regulatory networks.

This review systematically summarizes the structural characteristics and molecular functions of SIRT7, as well as its implications in human diseases. We summarize key advances in the field over the past five years, with a particular focus on recent discoveries regarding SIRT7 functions in the nervous system and their potential underlying mechanisms. Finally, we discuss current limitations and propose future research directions.

## 2. Structure and Molecular Mechanism of SIRT7

SIRT7 contains a conserved catalytic core domain responsible for NAD^+^ binding and deacylation activity. This core domain is composed of a Rossmann fold that accommodates NAD^+^ and a zinc-binding module formed by conserved cysteine residues. SIRT7 was initially regarded as a deacetylase; however, structural and biochemical analyses indicate that its intrinsic enzymatic activity is relatively weak compared with that of SIRT1 or SIRT6. Deacetylation by SIRT7 is highly dependent on the cellular context. Although SIRT7 activity can be stimulated by RNA and DNA under in vitro conditions [10,11], efficient and site-specific catalysis in vivo requires higher-order chromatin structures. Notably, SIRT7 deacetylates H3K36 and H3K18 exclusively within intact nucleosomes, where DNA architecture and histone–DNA geometry constrain substrate presentation and determine lysine accessibility [6].

In addition to deacetylating specific histone residues, SIRT7 displays broader lysine deacylation activities, suggesting its role as a versatile, context-dependent regulator of protein acylation states [11]. SIRT7 has been identified as a histone desuccinylase that targets histone H3 at lysine 122 (H3K122), thereby promoting chromatin compaction and maintaining genome stability [12]. Moreover, SIRT7 has been demonstrated to function as a histone deglutarylase, specifically catalyzing the removal of glutaryl groups from histone H4 at lysine 91 (H4K91), thus regulating chromatin dynamics [13]. Precise kinetic analyses have demonstrated that SIRT7 exhibits a strong preference for demyristoylation and depropionylation, and have identified NAD^+^ affinity as a key determinant of acyl substrate selectivity [14]. Beyond its lysine-directed deacylation activities, SIRT7 has been reported to possess an NAD^+^-independent deacetylase activity toward N4-acetylcytidine (ac4C) on ribosomal RNA [15]. The ac4C modification is known to regulate RNA stability and translational efficiency; therefore, deacetylation of rRNA ac4C by SIRT7 suggests a role in ribosome biogenesis, translational control, and the regulation of cellular protein synthesis capacity.

Taken together, SIRT7 is a multifunctional enzyme that exhibits multiple NAD^+^-dependent lysine deacylation activities as well as NAD^+^-independent RNA deacetylation activity, and plays important roles in chromatin regulation, protein acylation modification, and ribosome function. Further identification of additional substrates, functional sites, and regulatory mechanisms will be essential for a more comprehensive understanding of its biological roles and physiological significance.

In addition to the conserved catalytic core, the N- and C-terminal regions of SIRT7 show limited sequence homology to other sirtuins and contribute to its functional specificity. The N-terminal region is relatively short and enriched in basic amino acids, which may facilitate interactions with chromatin and nucleic acids. In contrast, the C-terminal region of SIRT7 is structurally distinct and plays a critical role in nucleolar localization, protein–protein interactions, and full enzymatic activity [16].

Notably, SIRT7 contains nucleolar localization signals that enable its preferential accumulation in the nucleolus, a feature that distinguishes it from most other sirtuin family members. This structural adaptation allows SIRT7 to directly associate with rDNA loci and RNA polymerase I-associated factors, thereby linking its structural organization to its central role in ribosomal RNA synthesis and processing [17]. The nucleolar localization of SIRT7 is dynamic and can be altered in response to metabolic stress or signaling cues. Under conditions such as DNA damage, nucleolar stress, or metabolic perturbations, SIRT7 can redistribute from the nucleolus to the nucleoplasm or chromatin-associated sites [18], further suggesting the functional importance of its structural elements.

Collectively, the structural features of SIRT7—including its conserved but context-dependent catalytic core, as well as its unique N- and C-terminal regions—provide the structural framework underlying its substrate selectivity and functional specialization. These characteristics distinguish SIRT7 from other sirtuins and underpin its emerging roles in tissue-specific biological processes, including those in the nervous system.

## 3. An Overview of Key Substrates of SIRT7

Extensive studies have characterized the deacetylation substrates of SIRT7, establishing that its major targets encompass both histone and non-histone proteins. These substrates display pronounced localization- and context-dependent regulation, reflecting their dynamic distribution between nucleolar and extranucleolar compartments.

SIRT7 function is not restricted to deacetylation but also exhibits a broader lysine deacylation capacity. This includes the removal of long-chain fatty acyl groups, such as demyristoylation and depropionylation, as well as other non-acetyl acyl modifications, including desuccinylation, deglutarylation, and decrotonylation [19]. Importantly, these non-acetyl deacylation activities are often more pronounced under specific activating conditions, such as RNA- or DNA-mediated activation or within distinct chromatin contexts, thereby expanding the substrate spectrum and functional scope of SIRT7.

SIRT7 exhibits dynamic subcellular shuttling and is primarily distributed within the nucleolus and nucleoplasm, with a minor fraction detectable in the cytoplasm [18]. In response to various forms of cellular stress—including nucleolar stress, genotoxic stress, and metabolic stress—SIRT7 undergoes marked relocalization or translocation, resulting in a corresponding switch in substrate targeting [17].

Deacylation of these substrates by SIRT7 not only directly modulates their intrinsic activity, stability, and protein–protein interactions, but also subcellular localization. This coordinated regulatory capacity provides a molecular basis for the emerging concept of SIRT7 as an important regulator of cellular homeostasis. We systematically summarize the major validated substrates of SIRT7, with particular emphasis on the mechanisms by which its deacetylation and other deacylation activities regulate substrate function, subcellular localization preference, regulatory signal, and downstream signaling pathways (Table 1), thereby laying the foundation for subsequent functional discussions.

## 4. Molecular and Biochemical Functions of SIRT7

### 4.1. rRNA Transcription and Processing

Within the nucleolus, SIRT7 mediates different cellular signals and targets a restricted but functionally critical set of substrates that collectively regulate ribosome biogenesis and nucleolar homeostasis.

Under growth-permissive conditions, SIRT7 localizes to rDNA loci in the nucleolus and interacts with components of the RNA polymerase I transcription machinery, including UBF. Through this interaction, SIRT7 facilitates the assembly of an active Pol I transcription complex and promotes pre-rRNA synthesis [21,31].

In contrast, nucleolar stress disrupts SIRT7 association with rDNA chromatin and results in repression of Pol I transcription. Mechanistically, SIRT7 regulates the acetylation state of the Pol I-associated factor PAF53, a critical component of the transcription machinery. Stress conditions inhibit SIRT7-dependent deacetylation of PAF53, leading to reduced Pol I activity and suppression of rDNA transcription. Consequently, rRNA synthesis and ribosome biogenesis are rapidly downregulated, representing an adaptive nucleolar stress response that limits cellular biosynthetic activity under unfavorable conditions [32].

Beyond stress signaling, SIRT7 also contributes to cell cycle-dependent control of rDNA transcription, ensuring high transcriptional output during interphase while enabling transcriptional silencing during mitosis. Mechanistically, SIRT7 deacetylates fibrillarin (FBL), thereby regulating its association with histone H2A and rDNA chromatin and influencing H2AQ104 methylation. During interphase, SIRT7 maintains FBL in a hypoacetylated, chromatin-bound state, supporting H2AQ104 methylation and robust RNA polymerase I transcription. By contrast, mitotic entry is accompanied by nucleolar disassembly and FBL hyperacetylation, which reduces chromatin binding, leads to loss of H2AQ104 methylation, promotes rDNA chromatin compaction, and ultimately represses Pol I-dependent transcription [22].

In addition to its role in transcriptional regulation, SIRT7 also controls pre-rRNA processing through its nucleolar substrate U3-55k, a core component of the U3 snoRNP complex. SIRT7-mediated deacetylation of U3-55k enhances its interaction with U3 snoRNA, which is essential for efficient pre-rRNA maturation. Under nucleolar stress or energy restriction, SIRT7 dissociates from the nucleolus, resulting in hyperacetylation of U3-55k and impaired pre-rRNA processing, thereby establishing a stress-sensitive SIRT7–U3-55k regulatory axis in ribosome biogenesis [33].

Collectively, SIRT7 integrates stress and cell cycle cues to regulate rDNA transcription and pre-rRNA processing through substrate-specific deacetylation. By modulating these transcriptional and ribosome biogenesis pathways, SIRT7 plays an important role in coordinating cellular growth and proliferation.

### 4.2. Chromatin Structure and Epigenetic Regulation

SIRT7 has emerged as a chromatin-associated sirtuin that primarily functions as a chromatin-compacting deacetylase, with a strong preference for histone H3 lysine 18 (H3K18) as its canonical substrate [20]. Extensive biochemical and structural studies demonstrate that SIRT7 selectively catalyzes H3K18 deacetylation, and Loss of H3K18ac leads to chromatin compaction, reduced recruitment of transcriptional coactivators, and impaired establishment of permissive marks such as H3K4me3, thereby restraining promoter- and enhancer-driven transcription [34]. Beyond H3K18, SIRT7 also regulates chromatin architecture through deacetylation of additional histone lysines. It acts as a robust deacetylase of H3K36/37, whose acetylation is associated with nucleosome loosening and transcriptional activation at transcription start sites; SIRT7-mediated removal of these acetylation groups is proposed to facilitate subsequent H3K36 methylation in a spatiotemporally controlled manner, contributing to heterochromatin formation and transcriptional repression [28]. Moreover, SIRT7 is recruited to DNA double-strand breaks in a PARP1-dependent manner, where it promotes chromatin condensation and genome stability by desuccinylating H3K122, a residue located at the nucleosomal dyad axis that directly affects DNA–histone interactions [12]. SIRT7 further controls chromatin compaction by acting as a deglutarylase for H4K91, a modification enriched at promoters of highly expressed genes and dynamically downregulated during mitosis and in response to DNA damage [13]. Although in vitro studies suggest that SIRT7 can also remove longer-chain acyl modifications, including propionyl and myristoyl groups—particularly at H3K18—the physiological relevance of these activities in vivo remains to be established [14].

Collectively, these findings position SIRT7 as an important epigenetic regulator that modulates multiple histone deacylation activities in response to various cellular contexts.

### 4.3. DNA Damage Repair and Genome Stability

SIRT7 functions as a genome integrity regulator by responding to DNA double-strand break (DSB) damage through histone deacetylation. Upon DNA damage, SIRT7 is recruited to DSB sites in a PARP1-dependent manner, where it catalyzes deacetylation of histone H3K18. This SIRT7-mediated reduction in H3K18 acetylation facilitates the recruitment of the DNA damage response factor 53BP1 to DSBs, thereby promoting efficient non-homologous end joining repair, indicating SIRT7-mediated H3K18 deacetylation as a critical epigenetic mechanism linking DNA damage sensing to DSB repair and genome maintenance [7]. Upon completion of DNA repair, SIRT7 deacetylates ATM, facilitating its dephosphorylation and functional inactivation, thereby terminating ATM-dependent checkpoint signaling and restoring chromatin homeostasis and cell-cycle progression [23].

In contrast, under DNA-damaging chemotherapy, ATM phosphorylates SIRT7 and promotes its subcellular redistribution, enabling SIRT7 to interact with and deacetylate the mismatch repair core protein MSH2. This leads to MSH2 destabilization, transient suppression of mismatch repair, induction of microsatellite instability, and enhanced adaptive mutagenesis, ultimately conferring chemotherapeutic resistance in cancer cells [24].

Beyond these roles in canonical DNA damage signaling, SIRT7 is also involved in the maintenance of genome integrity through its interaction with the RNA helicase DDX21, where SIRT7-mediated deacetylation of DDX21 suppresses R-loop accumulation and prevents transcription-associated DNA double-strand breaks at ribosomal DNA loci, thereby preserving nucleolar integrity and genomic stability [29]. Together, these findings establish SIRT7 as a multifaceted regulator that integrates DNA repair, transcriptional stress control, and genome stability in a highly context-dependent manner.

Overall, SIRT7 contributes to the maintenance of genome integrity by fine-tuning epigenetic marks, repair factor recruitment, and the timing of the DNA damage response, whereas its depletion has been associated with increased sensitivity to DNA damage and elevated mutagenesis risk.

### 4.4. Regulation of Transcription

Accumulating evidence indicates that SIRT7 is involved in regulating cell fate decisions by coordinating both global and gene-specific transcriptional programs. In addition to controlling global transcriptional output, SIRT7 directly interacts with and deacetylates lineage-determining transcription factors, thereby fine-tuning their transcriptional activity at specific target genes. Through these complementary mechanisms, SIRT7 shapes context-dependent gene expression programs that govern distinct cellular identities and states. Such regulatory functions are involved in diverse biological processes, including cardiac development, osteoblast differentiation, immune cell maturation, and stem cell state transitions, underscoring SIRT7 as a central modulator of transcriptional networks that integrate broad transcriptional control with precise gene-specific regulation of cell fate.

- Regulation of Global Transcription

In contrast to its role in promoting rDNA transcription, SIRT7 directly participates in the regulation of global transcription by modulating RNA polymerase II-dependent transcriptional elongation. SIRT7 has been shown to deacetylate and activate CDK9, the catalytic subunit of the positive transcription elongation factor b, which is required for productive transcription across the genome [26], thereby sustaining high transcriptional output required for proliferation, stress recovery, and tumorigenic growth.

SIRT7 also plays a role in transcriptional regulation through deacetylation-independent modulation of transcriptional machinery. SIRT7 physically associates with the transcription factor Myc and is recruited to ribosomal protein gene promoters, where it functions as a transcriptional corepressor to restrain Myc-driven global transcription. Importantly, current evidence indicates that this regulatory function does not involve direct deacetylation of Myc, underscoring a distinct mode of transcriptional control.

Through the integration of enzymatic and non-enzymatic mechanisms, SIRT7 serves as an important regulator of global transcriptional programs, enabling cells to adapt transcriptional output to developmental cues and cellular stress conditions [35].

- Regulation of Mitochondrial Gene Transcription

SIRT7 regulates mitochondrial gene transcription by modulating nuclear transcription factors involved in mitochondrial biogenesis and energy homeostasis. SIRT7 directly deacetylates GABPβ1, promoting its association with GABPα and formation of the active GABPα/GABPβ heterotetramer, thereby enhancing transcription of nuclear-encoded mitochondrial genes [27]. Although the upstream regulatory signals remain incompletely defined, this SIRT7–GABPβ1 axis is functionally linked to cellular energy status, suggesting that SIRT7 couples metabolic state to mitochondrial gene transcription through post-translational control of GABPβ1.

- Regulation of Specific Gene Transcription

SIRT7 directly interacts with multiple DNA-binding transcriptional factors and fine-tunes their transcriptional output through site-specific deacetylation. In the cardiovascular system, SIRT7 deacetylates the cardiac transcription factor GATA4 at lysine 311, thereby suppressing its transcriptional activity and limiting the expression of hypertrophic and angiogenic target genes such as ANP, BNP, and VEGFA. SIRT7 acts as a negative regulator of cardiac transcriptional programs involved in heart development and pathological remodeling [36]. In ovarian cancer cells, SIRT7 directly deacetylates GATA4, resulting in repression of its transcriptional activity. Loss of GATA4 function consequently activates Wnt signaling, thereby driving cancer cell proliferation and tumor progression [37]. Therefore, the biological consequences of SIRT7-dependent GATA4 deacetylation are highly tissue-specific. In skeletal biology, SIRT7 directly deacetylates Osterix (OSX/SP7), a master transcription factor essential for osteoblast differentiation and bone formation, at lysine 368. This modification facilitates subsequent depropionylation by SIRT1 and enhances the N-terminal transactivation activity of OSX. Through this coordinated regulation of post-translational acylation, SIRT7 activity is associated with osteoblast differentiation and maintains bone homeostasis [38].

Moreover, SIRT7 regulates gene expression through the modulation of transcription factor stability, impacting multiple cellular processes. Specifically, deacetylation of Pax5 at lysine 198 by SIRT7 enhances its protein stability and transcriptional activity, ensuring proper B cell lineage commitment, differentiation, and maturation [39]. Conversely, SIRT7 negatively regulates NFATc1-dependent transcription by deacetylating NFATc1 at lysine 612, promoting its proteasomal degradation. This inactivation of NFATc1 facilitates exit from quiescence and promotes hair regeneration [40]. Beyond stem cell regulation, NFATc1 functions as a master transcription factor integrating immune activation [41] and skeletal homeostasis [42] through a distinct gene expression program, suggesting that SIRT7 may modulate immune responses and osteoclast differentiation through regulation of NFATc1, although the in vivo relevance of this axis requires further investigation.

SIRT7 also exerts epigenetic control and functions as a transcriptional repressor of a defined subset of genes. Through interaction with the ETS family transcription factor ELK4, SIRT7 is recruited to target gene promoters, where it mediates H3K18 deacetylation, resulting in transcriptional silencing of tumor suppressor genes and promotion of oncogenic growth [20]. Another study has shown that SIRT7 is involved in the assembly of epigenetic regulatory complexes by directly deacetylating WDR77 at lysine residues 3 and 243, weakening its association with the PRMT5 methyltransferase complex and reducing symmetric arginine dimethylation of histones and other substrates. This impairment of PRMT5-dependent epigenetic activity suppresses cancer cell proliferation, exemplified in colorectal cancer models [43].

Collectively, these findings establish SIRT7 as a context-dependent transcriptional rheostat that orchestrates cell fate decisions across diverse tissues by coupling deacetylation of key transcriptional regulators to developmental, homeostatic, and regenerative programs.

### 4.5. Regulation of the p53 Signaling Network by SIRT7

SIRT7 regulates p53 signaling through multiple mechanisms in a context-dependent manner. Under basal or tumor-promoting conditions, SIRT7 suppresses p53 activity both directly and indirectly. SIRT7 physically interacts with p53 and promotes its deacetylation, reducing its binding to the promoter of the pro-apoptotic gene NOXA and attenuating p53-dependent apoptosis [25].

In addition, SIRT7 indirectly modulates p53 stability through deacetylation of the adaptor protein STRAP, which enhances p53 ubiquitination and decreases its transcriptional activity [44]. SIRT7 also negatively regulates ARF protein stability, further weakening ARF-mediated activation of the p53 tumor suppressor pathway [45].

In contrast, under genotoxic stress SIRT7 promotes p53 stabilization through nucleolar stress signaling. Upon UV-induced DNA damage, ATR activates SIRT7, enabling deacetylation of the nucleolar protein NPM. Deacetylated NPM relocates to the nucleoplasm, where it inhibits MDM2-mediated p53 ubiquitination, thereby stabilizing p53 and promoting downstream stress responses such as cell-cycle arrest and apoptosis [46].

Thus, SIRT7 exerts context-dependent effects on p53, either suppressing or stabilizing its activity depending on cellular conditions.

### 4.6. Critical Signaling Regulators Targeted by SIRT7

One important functional category of SIRT7 substrates involves regulators of developmental and metastatic signaling. SIRT7 directly deacetylates SMAD4, promoting its β-TrCP1-mediated ubiquitination and proteasomal degradation. Loss of SMAD4 stability attenuates transforming growth factor-β (TGF-β) signaling, thereby suppressing epithelial-to-mesenchymal transition (EMT) and inhibiting breast cancer metastasis, establishing SIRT7 as a negative regulator of TGF-β-driven tumor dissemination [47].

In addition to transcriptional regulators, SIRT7 targets metabolic enzymes and signaling adaptors to reprogram cellular metabolism and growth signaling. SIRT7 deacetylates phosphoglycerate kinase 1 (PGK1), a key glycolytic enzyme whose acetylation enhances enzymatic activity and supports cancer cell proliferation and tumorigenesis. By modulating PGK1 acetylation, SIRT7 directly links post-translational regulation to glycolytic flux and metabolic support of tumor growth [48]. Furthermore, SIRT7 deacetylates the scaffold protein FKBP51 at lysines 28 and 155, strengthening its interaction with Akt and the phosphatase PHLPP, thereby promoting Akt dephosphorylation. This mechanism becomes particularly pronounced under energy stress, revealing a role for SIRT7 in integrating metabolic stress with Akt-dependent survival signaling [49].

Collectively, SIRT7 either activates or represses diverse signaling and metabolic pathways through the deacetylation of critical transcriptional regulators and signaling mediators, thereby fine-tuning stress responses and context-specific signal transduction.

## 5. Physiological and Pathological Roles of SIRT7

### 5.1. Immune Responses

SIRT7 has been implicated in immune regulation by targeting key substrates involved in innate immune sensing, inflammatory transcription, and adaptive immune cell development. It regulates inflammatory responses by controlling nucleocytoplasmic transport. It deacetylates the small GTPase Ran, inhibiting its acetylated form and reducing its interaction with the nuclear export receptor CRM1. This prevents the nuclear export of NF-κB subunit p65, enhancing its nuclear retention and transcriptional activity. As a result, SIRT7 influences NF-κB-dependent inflammatory and stress-related gene expression, particularly in inflammation and cancer [50]. Beyond acute inflammatory signaling, SIRT7 contributes to innate immune homeostasis by preserving heterochromatin integrity and suppressing aberrant activation of transposable elements. SIRT7 deficiency leads to derepression of LINE1 retrotransposons, which in turn activates the cGAS–STING pathway and triggers innate immune signaling. This mechanism is particularly relevant during aging, as declining SIRT7 expression in human mesenchymal stem cells promotes chronic inflammatory signaling and accelerates cellular senescence [51]. SIRT7 also exerts essential functions in adaptive immunity by regulating B cell lineage specification. Through direct deacetylation of the B cell-specific transcription factor Pax5 at lysine 198, SIRT7 enhances Pax5 protein stability and transcriptional activity, thereby ensuring proper B cell lineage commitment, early differentiation, and maturation [39]. Collectively, these findings establish SIRT7 as a multifaceted regulator of immune homeostasis, coordinating innate immune restraint, inflammatory transcriptional control, and adaptive immune development through substrate-specific deacetylation mechanisms.

### 5.2. Stem Cell Maintenance and Aging

SIRT7 has been implicated in the regulation of hematopoietic stem cell (HSC) fate and lineage differentiation during aging and stress conditions. Mechanically, SIRT7 interacts with the transcription factor NRF1 to repress the expression of mitochondrial ribosomal and translational genes, thereby limiting mitochondrial protein folding stress and restraining mitochondrial unfolded protein response activation. This regulatory axis helps maintain low mitochondrial activity and preserves HSC quiescence [52]. In line with this mechanistic framework, studies using a mouse model with Sirt7 deletion in hematopoietic stem cells demonstrated that Sirt7 suppresses abnormal HSC differentiation in aged mice, thereby maintaining hematopoietic homeostasis [53]. The underlying molecular mechanisms, however, remain to be further elucidated.

Beyond its role in hematopoietic stem cells, emerging evidence indicates that SIRT7 also regulates stem cell behavior in other tissue compartments. SIRT7 activates quiescent hair follicle stem cells to ensure hair growth in mice. Sirt7 is downregulated in aged HFSCs, and exogenous Sirt7 overexpression promotes hair growth in aged animals [40].

Collectively, these findings establish SIRT7 as a context-dependent regulator of stem cell homeostasis across multiple tissues. Notably, SIRT7 expression declines with aging in both HSCs and HFSCs, and this age-associated reduction contributes to impaired stem cell function and diminished tissue regenerative capacity. Together, these observations indicate an important role for SIRT7 in preserving stem cell fitness and adaptive potential across distinct stem cell compartments during aging.

### 5.3. Circadian

Emerging evidence suggests that SIRT7 participates in circadian rhythm regulation by modulating the stability of core clock components. It directly targets Cryptochrome 1 (CRY1), a central repressor within the molecular circadian clock and deacetylates CRY1 at specific lysine residues, including K565 and K579, which promotes FBXL3-mediated ubiquitination and proteasomal degradation of CRY1. By controlling CRY1 protein stability, SIRT7 fine-tunes circadian phase coherence in the liver and ensures proper oscillation of clock-controlled metabolic genes. Functionally, this regulatory mechanism links SIRT7 activity to rhythmic hepatic gluconeogenesis and maintenance of glucose homeostasis, indicating SIRT7 as a regulator connecting NAD^+^-dependent deacetylation, circadian timing, and metabolic regulation [54]. However, studies investigating the role of SIRT7 in circadian regulation remain limited. Future investigations should therefore determine whether SIRT7 exerts circadian regulatory functions in other metabolically active tissues, such as skeletal muscle, adipose tissue, and pancreas, where tight coupling between the molecular clock and metabolism is essential for systemic homeostasis. In parallel, it will be important to explore whether SIRT7 contributes to circadian control within the central nervous system, particularly in the suprachiasmatic nucleus, and whether it plays a role in modulating the master circadian pacemaker.

### 5.4. Oxidative Stress and Ferroptosis Regulation

SIRT7 has been implicated in the regulation of cellular redox homeostasis by deacetylating key components of the KEAP1–NRF2 antioxidant axis. It directly deacetylates NRF2, reducing its binding to the cytoplasmic repressor KEAP1, which enhances NRF2 stability and promotes its nuclear translocation [55]. In parallel, SIRT7 deacetylates KEAP1, weakening its ability to sequester NRF2, further facilitating the dissociation of the KEAP1–NRF2 complex [56]. Together, these modifications enhance NRF2 activity and facilitate the transcriptional activation of antioxidant genes, thereby maintaining intracellular redox balance. Furthermore, SIRT7 is involved in the regulation of ferroptosis under oxidative stress conditions. Mechanistically, SIRT7 deacetylates SMAD3 and suppresses its transcriptional activity, which in turn downregulates ATF3 expression. Because ATF3 acts as a negative regulator of GPX4, reduced ATF3 relieves repression on GPX4, leading to increased GPX4 expression. Elevated GPX4 efficiently detoxifies lipid peroxides, thereby limiting lipid peroxidation and protecting cells from ferroptotic death [57]. These findings suggest that SIRT7 may regulate multiple signaling pathways involved in oxidative stress responses. Together, these observations raise the possibility that SIRT7 possesses a broader substrate spectrum and spatial regulatory capacity than previously appreciated.

### 5.5. Context-Dependent Function of SIRT7 in Cancer

**Figure 1:**
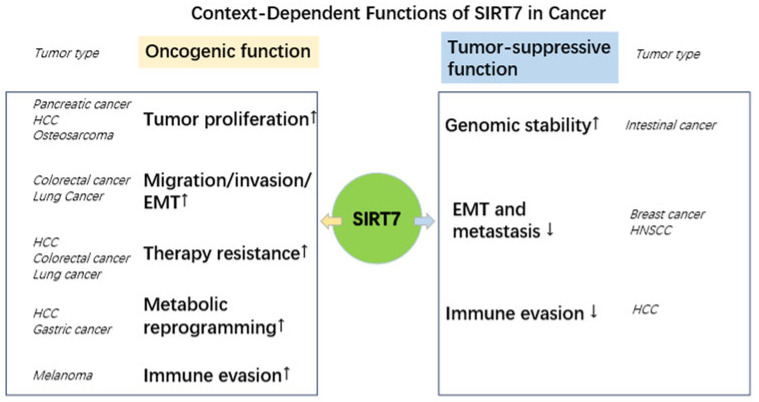
Context-dependent functions of SIRT7 in cancer. This schematic illustrates the diverse functions of SIRT7 across multiple cancers. The left panel indicates oncogenic roles of SIRT7, whereas the right panel represents tumor-suppressive functions. Arrows (“↑”) indicate increased effect, arrows (“↓”) indicate decreased effect. Abbreviations: HCC, hepatocellular carcinoma, HNSCC, head and neck squamous cell carcinoma; EMT, epithelial–mesenchymal transition.

SIRT7 plays different roles in cancer in a context-dependent manner. In different tumor types, different development stages, and different stress conditions, SIRT7 can promote tumor growth, drug resistance, and immune evasion through distinct signaling pathways, while in some cases it can also show tumor-suppressive effects (shown in Figure 1). The detailed information is provided in the following paragraphs.

#### 5.5.1. Oncogenic Roles of SIRT7

SIRT7 has been shown to enhance tumor growth through a dual mechanism involving epigenetic chromatin modification and G1/S cell-cycle progression. In pancreatic cancer [58], hepatocellular carcinoma [59], fibrosarcoma [20], and osteosarcoma [60], SIRT7 deacetylates histone H3K18ac, leading to epigenetic silencing of tumor suppressor genes and enhanced proliferative capacity. In addition, in several digestive system cancers, SIRT7 facilitates cell-cycle progression by upregulating Cyclin D1 and repressing the CDK inhibitor p21, thereby accelerating the G1/S transition [61].

SIRT7 also contributes to tumor invasiveness by activating major oncogenic signaling cascades that drive EMT. In colorectal cancer, SIRT7 enhances the Raf–MEK–ERK MAPK pathway, thereby promoting EMT and increasing migratory and metastatic potential [62]. In lung cancer, SIRT7 drives malignant progression through coordinated activation of AKT and ERK1/2 signaling, supporting both G1/S cell-cycle progression and reinforcing EMT-associated phenotypes [63]. These observations suggest that SIRT7 regulates tumor migration and metastasis through amplification of pro-migratory signaling networks.

Moreover, multiple studies suggest SIRT7 enhances tumor cell survival and therapy resistance. In hepatocellular carcinoma, SIRT7 deacetylates and functionally suppresses p53, attenuating p53-dependent apoptotic signaling and increasing resistance to anticancer drugs [25]. In colorectal cancer, SIRT7 increases cell viability following combined 5-fluorouracil and radiotherapy treatment, thereby promoting therapy tolerance [64]. In lung cancer, SIRT7 facilitates gemcitabine-induced protective autophagy, further supporting drug resistance [65]. Phosphorylation of SIRT7 following treatment with purine analogs disrupts the mismatch repair pathway, facilitating adaptive mutagenesis and chemoresistance in cervical and lung cancer cells [24]. Together, these findings indicate that SIRT7 enables tumor cells to adapt to cytotoxic therapies by repressing apoptotic programs and activating stress-protective pathways.

SIRT7 is involved in metabolic reprogramming and growth signaling in tumors. In hepatocellular carcinoma, SIRT7 promotes lipid metabolic reprogramming through desuccinylation of PRMT5, enhancing PRMT5-dependent methylation of SREBP1a and driving tumor growth and metastasis (Yuan et al., 2022) [30]. In gastric cancer, SIRT7 activates the mTOR–IGF2 signaling axis, suppressing apoptosis and autophagy to support tumor cell survival (Yu et al., 2018) [66]. These studies collectively support that SIRT7 is involved in metabolic remodeling and pro-survival signaling in cancer cells.

Emerging evidence suggests that SIRT7 contributes to the regulation of tumor immunity in a cancer-type-dependent manner. In melanoma, SIRT7 deacetylates and destabilizes SMAD4, activating the IRE1α–XBP1 pathway and inducing PD-L1 expression, thereby promoting immune evasion [67]. This finding suggests that SIRT7 inhibition may increase the efficacy of anti-PD-L1 immunotherapy.

#### 5.5.2. Tumor-Suppressive Functions of SIRT7

It has been reported SIRT7 exerts a tumor-suppressive function during early tumorigenesis by maintaining genomic stability, particularly in the context of intestinal cancer. SIRT7 preserves rDNA heterochromatin integrity and prevents recombination of repetitive rDNA sequences [68]. In murine models of intestinal tumorigenesis, SIRT7 deficiency leads to reduced HAT1 activity, centrosome-associated chromatin instability, and aneuploidy, thereby accelerating tumor initiation [69].

In breast cancer and HNSCC, SIRT7 suppresses EMT and metastasis through regulation of the TGF-β/SMAD4 signaling axis [47,70]. In these tumor types, reduced SIRT7 expression is associated with increased metastatic potential.

Studies have shown that in hepatocellular carcinoma (HCC), SIRT7 functions as a positive regulator of anti-tumor immunity by suppressing PD-L1 expression. Mechanistically, SIRT7 deacetylates the transcription factor MEF2D, reducing its recruitment to the CD274 (PD-L1) promoter and thereby downregulating PD-L1 transcription [71]. Through this pathway, SIRT7 enhances immune recognition and cytotoxic clearance of tumor cells. Notably, this immune-modulatory role contrasts with its pro-immune-escape function in melanoma, further emphasizing the cancer-type-dependent nature of SIRT7-mediated immune regulation.

Collectively, current evidence highlights the multifaceted and context-dependent roles of SIRT7 in tumor biology. These seemingly contradictory findings indicate that the biological functions of SIRT7 are strongly influenced by tumor type, cellular context, and the signaling pathways involved. Therefore, a deeper understanding of the context-specific regulation and downstream targets of SIRT7 will be essential for clarifying its dual roles and for evaluating its potential as a therapeutic target in cancer.

## 6. Functions of SIRT7 in Nervous System

Although extensive research has focused on the role of SIRT7 in tumor regulation—where it often exhibits context-dependent oncogenic or tumor-suppressive functions, its potential roles in other physiological systems, including the nervous system, are only beginning to be explored.

SIRTUIN family proteins are widely expressed in various cell types of the central nervous system (CNS), such as neurons, astrocytes, and microglia. Different members exhibit specific subcellular localizations: SIRT1 and SIRT2 are primarily nuclear and/or cytoplasmic, SIRT3–SIRT5 are localized in mitochondria, and SIRT6 and SIRT7 are mainly nuclear. Through deacetylation of histones and non-histone proteins, they regulate synaptic plasticity, calcium homeostasis, circadian rhythms, and anti-aging processes [72]. SIRT1, SIRT3, and SIRT6 serve as core members for neuroprotection, which reduce neuroinflammation, regulate mitochondrial biogenesis, and enhance autophagy and exert clear protective effects in diseases including Alzheimer’s disease, Parkinson’s disease, Huntington’s disease, amyotrophic lateral sclerosis, ischemic injury, and other forms of neural trauma [73,74,75,76].

**Figure 2:**
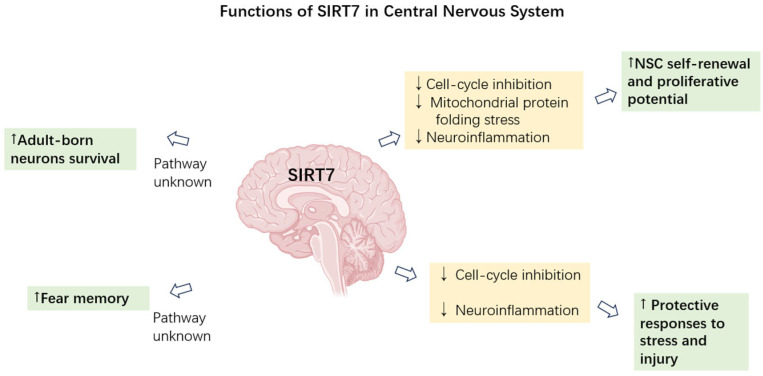
Functions of SIRT7 in central nervous system. This schematic illustrates the recent understanding of functions of SIRT7 and the related mechanisms in the central nervous system. Green regions indicate the outcome of SIRT7 activities in neuron system, and beige regions represent pathways involved. Arrows (“↑”) indicate increased effect, arrows (“↓”) indicate decreased effect. Brain illustration created with BioRender.com.

SIRT1 and SIRT6 exert neuroprotective effects through multiple mechanisms, including promoting DNA damage repair, enhancing resistance to oxidative stress, suppressing neuroinflammatory responses, regulating cellular metabolism and mitochondrial function, and maintaining genomic stability [77,78]. SIRT7 has been linked to the regulation of transcriptional programs, cellular stress responses, and inflammatory processes, all of which are critical determinants of neuronal survival and plasticity. These observations provide a strong rationale for investigating the potential functions of SIRT7 in the nervous system, especially under conditions of inflammation or cellular stress. In recent years, important discoveries have begun to emerge, revealing potential neuroprotective roles for SIRT7 in contexts such as cerebral ischemia, neuroinflammation, and adult neurogenesis. In this section, we summarize key recent findings on SIRT7’s functions in the nervous system (shown in Figure 2) and detailed information is provided in the following paragraphs.

### 6.1. Immune Regulation and Survival of Hippocampal Neurons

Emerging evidence indicates that SIRT7 may play a role in immune regulation and the survival of newly generated hippocampal neurons. SIRT7 deficiency was associated with mild immune alterations and reduced survival of newly generated neurons in the hippocampus. At the molecular level, SIRT7 is proposed to exert its effects through epigenetic regulatory mechanisms, including modulating inflammatory responses and intracellular stress-related transcriptional programs [79]. These findings provide preliminary evidence suggesting that SIRT7 may contribute to neural plasticity and adult neurogenesis through pathways linked to stress and inflammation.

### 6.2. CNS Function

SIRT7 is involved in higher-order central nervous system functions. SIRT7 has been shown to be involved in the consolidation of fear memory in mice. As no overt developmental or structural abnormalities were observed in the brains of *Sirt7* knockout mice, the impact of SIRT7 on the central nervous system is more likely mediated at the functional or molecular regulatory level rather than through gross neurodevelopmental defects. Importantly, this work represents the first study to extend the characterization of SIRT7 from a broadly characterized cellular and metabolic regulator to a specific central nervous system functional phenotype [80].

### 6.3. Neural Stem Cell Regulation

SIRT7 has been implicated in the regulation of hippocampal neural stem cell (NSC) proliferation and maintenance. In mouse hippocampal NSCs, SIRT7 functions as a critical pro-proliferative regulator by actively repressing the cell-cycle inhibitor p21, thereby sustaining NSC self-renewal and proliferative potential. Under glucocorticoid-induced stress (e.g., dexamethasone, DEX), activation of glucocorticoid receptor signaling suppresses SIRT7 expression, which in turn abolishes SIRT7-mediated restraint of p21. Consequently, p21 becomes upregulated and enforces cell-cycle inhibition, ultimately leading to impaired NSC proliferation (Alnoud, Chen et al., 2021) [81].

Recent studies suggest that mitochondrial protein folding stress is a key driver of hippocampal NSC aging, which can subsequently contribute to neural hyperactivity and cognitive decline. SIRT7 is proposed to suppress mitochondrial protein folding stress by inhibiting the activity of the transcription factor NRF1 and is thereby involved in maintaining NSC homeostasis and sustaining adult hippocampal neurogenesis. Notably, lentivirus-mediated SIRT7 overexpression in the dentate gyrus of aged mice has been shown to enhance neurogenesis and improve learning and memory performance, suggesting that modulation of SIRT7 activity may influence age-related neurogenic and cognitive processes [82].

However, a critical mechanistic gap remains unresolved: it is still unclear whether SIRT7 deacetylase activity in NSCs is essential to repress the NRF1-mediated transcriptional program. Elucidating this mechanism would not only strengthen the causal framework linking mitochondrial proteostasis to NSC aging and cognitive dysfunction, but also directly facilitate the identification of molecular targets and biomarkers within the SIRT7–NRF1 regulatory axis.

### 6.4. Stress and Injury Responses

It has been shown that SIRT7 participates in the regulation of ischemic brain injury through multiple cellular and molecular pathways. In an in vitro oxygen–glucose deprivation and reoxygenation (OGD/R) model, SIRT7 overexpression was shown to be associated with reduced cell death. These effects were reported to be associated with repressing the p53 signaling pathway, a central regulator of stress-induced apoptosis in ischemic conditions [83]. Thus, the SIRT7–p53 axis as a pathway regulating neuron cell death requires further investigation.

It has been shown that, after cerebral ischemia or OGD, restoring SIRT7 expression reduces infarct volume, brain edema, neuronal apoptosis, and pro-inflammatory cytokine production in microglia. Mechanistically, SIRT7 directly interacts with nicotinamide phosphoribosyltransferase (NAMPT) and promotes NAMPT desuccinylation, which decreases NAMPT stability via proteasomal degradation; this downregulation of NAMPT suppresses microglial M1 polarization while promoting M2 polarization, ultimately attenuating neuroinflammation-driven secondary neuronal damage [84].

Despite these emerging findings, most evidence is derived from mouse models or in vitro systems, and validation in human neural tissues remains limited. Moreover, while SIRT7 has been associated with neurogenesis, NSC regulation, memory processes, and stress responses, the underlying molecular mechanisms are not yet fully established. In particular, whether pathways such as NRF1-mediated mitochondrial regulation or p53 signaling represent direct targets of SIRT7 or indirect downstream effects remains unclear. Therefore, many proposed functions of SIRT7 in the brain should be considered preliminary and require further investigation in cell-type-specific and in vivo models.

## 7. Future Directions for SIRT7 Research in the Nervous System

### 7.1. Neuroenergetic Regulation and Cell-Type-Specific Metabolic Adaptation

Neural cell types display distinct metabolic dependencies: neurons rely heavily on mitochondrial oxidative phosphorylation to sustain activity-dependent ATP demands, whereas microglia exhibit metabolic plasticity, shifting toward glycolysis in pro-inflammatory states and toward oxidative phosphorylation and fatty acid oxidation in reparative states [85]. As an NAD^+^-dependent deacylase, SIRT7 is well positioned to sense energetic stress and metabolic fluctuations, yet its cell-type-specific functions in the brain remain largely undefined.

One of the key pathological features of Parkinson’s disease (PD) is the progressive degeneration of dopaminergic neurons in the substantia nigra pars compacta (SNpc), accompanied by reduced dopamine signaling in the striatum. Mitochondrial dysfunction, oxidative stress, and metabolic dysregulation are widely considered central contributors to PD pathogenesis [85].

Using PD as a disease context, the nigrostriatal system can serve as an informative model to investigate SIRT7-regulated protein modifications. By employing SIRT7 wild-type and knockout mice, LC–MS/MS-based acetylome and acylome profiling in the SNpc and striatum may enable the identification of novel SIRT7 substrates and lysine modification sites, potentially including acyl modifications beyond acetylation [14,86]. Integrating proteomic, metabolomic, and functional analyses may further reveal whether these modification changes are associated with pathways involved in mitochondrial metabolism, redox homeostasis, and dopamine metabolism, thereby providing insights into the potential link between SIRT7-mediated deacylation and the selective vulnerability of dopaminergic neurons in PD.

Future work should map SIRT7 substrates and deacylation targets across neuronal subtypes and glia, and determine how SIRT7 integrates metabolic stress with redox and inflammatory responses in physiological and disease contexts.

### 7.2. Genome Stability, Nucleolar Stress, and Neurodegeneration

Accumulation of DNA damage is a hallmark of aging and neurodegenerative disease. Because neurons are long-lived and post-mitotic, impaired DNA repair contributes to transcriptional dysregulation, synaptic dysfunction, and progressive neuronal loss [87,88]. Declining repair efficiency further promotes genomic instability and chronic neuroinflammation in both neurons and glial cells [89]. SIRT7 is an established regulator of genome integrity, participating in double-strand break repair and mismatch repair pathways [24,68]. In addition, it contributes to the maintenance of rDNA stability and nucleolar function [90]. However, the roles of SIRT7 in maintaining genome stability in post-mitotic neurons and glial cells in vivo remain largely unexplored.

Future studies should employ cell-type-specific Sirt7 conditional knockout mouse models combined with in vivo DNA damage paradigms and time-resolved repair assays to determine the function of SIRT7 in this process. Repair efficiency can be assessed by monitoring γH2AX/53BP1 dynamics, comet assays, and chromatin changes at damage sites, whereas downstream consequences can be evaluated through neuronal survival assays [91] and glial genome instability/inflammatory readouts, including cGAS-STING activation [92]. Rescue with wild-type versus catalytically inactive SIRT7 will be essential to establish whether SIRT7 enzymatic activity is required for these effects in vivo. To elucidate the role of SIRT7 in the nervous system may deepen our understanding of how DNA damage contributes to neurodegenerative disease pathogenesis and potentially inform the development of novel neuroprotective strategies.

### 7.3. Proteostasis and Stress-Adaptive Control of the Ubiquitin–Proteasome System

Proteostasis is central to neuronal survival, and age-related decline of the ubiquitin–proteasome system (UPS) is strongly linked to neurodegenerative pathology [93,94]. Notably, SIRT7 shows substrate- and context-dependent effects on protein degradation: it can promote quality-control turnover of misfolded proteins (e.g., via STUB1-dependent degradation of ERα) [95], but can also stabilize substrates by modulating E3 ligase complexes, such as the DCAF1/DDB1/CUL4B complex [96]. Moreover, SIRT7 has been proposed to act as a nucleolar stress sensor that regulates CRL4 activity via DDB1 deacetylation, thereby limiting proteasomal degradation of proteins such as p27, LATS1, and p73 and promoting stress tolerance [97]. However, whether these SIRT7-dependent UPS regulatory mechanisms operate in neurons or glial cells remains largely unknown. An important future direction will be to systematically identify SIRT7 substrates in neurons and glia and determine whether SIRT7 regulates their proteostasis through substrate-specific effects on ubiquitination, proteasomal turnover, or aggregation. This could be addressed using integrative genetic and proteomic approaches, including cell-type-specific manipulation of SIRT7 combined with quantitative acetylome/acylome and ubiquitinome profiling, as well as interactome mapping to identify candidate substrates. These targets can then be validated through ubiquitination and protein turnover assays. Their functional relevance should further be examined under proteotoxic stress conditions or in aggregation-prone disease models to determine whether SIRT7 influences protein aggregation, clearance, and neuronal survival.

Elucidating SIRT7-dependent regulation of proteostasis in neurons and glia may provide insight into how protein quality control is maintained during aging and stress in the nervous system. Because impaired UPS function and protein aggregation are hallmarks of neurodegenerative diseases, identifying SIRT7 substrates may reveal new links between various stress conditions and proteostasis and suggest potential therapeutic strategies.

### 7.4. Pharmacological Targeting of SIRT7

Given the potential therapeutic roles of SIRT7 in a variety of diseases, pharmacological targeting of SIRT7 is emerging as an important research direction. Although selective small-molecule modulators of SIRT7 remain limited, studies on compounds targeting other members of the sirtuin family demonstrate that selective pharmacological modulation of individual sirtuins is feasible. For instance, the SIRT1 activator SRT2104 and the SIRT6 activator MDL-800 illustrate that small molecules can selectively enhance the enzymatic activity of specific sirtuins [98,99]. These findings provide a conceptual framework for the development of SIRT7-targeted therapeutics. Therefore, the development of selective SIRT7 activators or inhibitors may not only enable modulation of key SIRT7-regulated pathways but also provide valuable tools for elucidating the biological functions of SIRT7.

However, translating SIRT7-targeting strategies to neurological disorders presents several central nervous system-specific challenges. Among the most critical are the efficient delivery of compounds across the blood–brain barrier and achieving sufficient target engagement and functional modulation in neurons and glial cells. Consequently, future studies will require not only the development of highly selective SIRT7 modulators but also advances in drug delivery strategies and CNS-targeting approaches. Addressing these challenges will be essential for evaluating the therapeutic potential of SIRT7 modulation in neurodegenerative diseases.

## 8. Summary

Collectively, studies in aging, inflammation, and cancer have established SIRT7 as a critical regulator of cellular homeostasis, capable of modulating proliferation, survival, and context-dependent invasive programs. These mechanistic findings provide a conceptual framework for extending SIRT7 research into the nervous system. Recent advances increasingly indicate that SIRT7 may contribute to the maintenance of neural integrity, with evidence supporting its involvement in neuronal survival and stress adaptation. However, the precise molecular mechanisms underlying SIRT7 function in neural cells remain incompletely understood. Future studies should focus on elucidating the underlying signaling pathways and cell-type-specific functions of SIRT7 in the central nervous system. Such efforts may provide new insights into the pathogenesis of brain aging, neurodegenerative disorders, and neural injury, and may help guide the development of SIRT7-targeted therapeutic strategies.

## Figures and Tables

**Table 1:** Key Substrates of SIRT7.

Representative Substrate	Subcellular Localization	SIRT7’s Effects	Outcome	Regulatory Signal	Reference(s)
H3K18	Nucleolus/rDNA chromatin	Deacetylation	↑Chromatin compaction, ↓rDNA Transcription	Nucleolar activity	[20]
H3K18	Nucleoplasm/DNA damage foci	Deacetylation	↑Recruitment of the DNA damage response factors and DNA repair	DNA damage	[7]
UBF	Nucleolus/rDNA chromatin	No modification reported	↑RNA Pol I activity And pre-rRNA synthesis	Nucleolar activity	[21]
Fibrillarin	Nucleus/rRNA processing	Deacetylation	↑RNA Pol I activity And pre-rRNA synthesis	Cell cycle and nucleolar activity	[22]
ATM	Nucleoplasm/DSB	Deacetylation	↓ATM activity and DNA damage response	DNA damage (Late stage)	[23]
MSH2	Nucleoplasm	Deacetylation	↓MMR activity	DNA damage	[24]
p53	Nucleoplasm	Deacetylation	↓Apoptosis and cell cycle arrest	DNA damage and oxidative stress	[25]
CDK9	Nucleoplasm	Deacetylation	↑RNA polymerase II and transcription elongation	Not reported	[26]
GABPβ1	Cytoplasm	Deacetylation	↑Transcription of nuclear-encoded mitochondrial genes	Cellular energy status	[27]
H3K36	Chromatin/specific promoter region	Deacetylation	↑Heterochromatin formation and transcription activity	Chromatin architecture/nucleosome structure	[28]
DDX21	Nucleolus-	Deacetylation	↓R-loop accumulation And transcription-associated DNA damage	Nucleolar activity	[29]
PRMT5	Nucleoplasm	Desuccinylation	↑Metabolic reprograming and tumor cell growth	Not reported	[30]
H3K122	Nucleoplasm	Desuccinylation	↑Chromatin condensation and genome stability	DSBs and PARP1 dependent	[12]
H4K91	Nucleoplasm	Deglutarylation	↑Chromatin condensation ↓Transcription activity	Cell cycle activity and DNA damage	[13]

Note: Arrows (“↑”) indicate increased effect, arrows (“↓”) indicate decreased effect. Abbreviations: DSB, Double-strand break.

## Data Availability

No new datasets were generated or analyzed in this study. Data sharing is not applicable to this article.
